# Molecular Characterization of *MaCCS*, a Novel Copper Chaperone Gene Involved in Abiotic and Hormonal Stress Responses in *Musa acuminata* cv. Tianbaojiao

**DOI:** 10.3390/ijms17040441

**Published:** 2016-03-24

**Authors:** Xin Feng, Fanglan Chen, Weihua Liu, Min Kyaw Thu, Zihao Zhang, Yukun Chen, Chunzhen Cheng, Yuling Lin, Tianchi Wang, Zhongxiong Lai

**Affiliations:** Institute of Horticultural Biotechnology, Fujian Agriculture and Forestry University, Fuzhou 350002, China; fengxin1506@163.com (X.F.); 13459404515@163.com (F.C.); 13559216985@163.com (W.L.); mktstar@gmail.com (M.K.T.); zhangzihao863@126.com (Z.Z.); cyk68@163.com (Y.C.); ld0532cheng@126.com (C.C.); buliang84@163.com (Y.L.); tianchi.wang@plantandfood.co.nz (T.W.)

**Keywords:** *Musa acuminata* cv. Tianbaojiao, *MaCCS*, promoter, abiotic stress, hormonal treatment, expression analysis

## Abstract

Copper/zinc superoxide dismutases (Cu/ZnSODs) play important roles in improving banana resistance to adverse conditions, but their activities depend on the copper chaperone for superoxide dismutase (CCS) delivering copper to them. However, little is known about *CCS* in monocots and under stress conditions. Here, a novel *CCS* gene (*MaCCS*) was obtained from a banana using reverse transcription PCR and rapid-amplification of cDNA ends (RACE) PCR. Sequence analyses showed that *MaCCS* has typical CCS domains and a conserved gene structure like other plant *CCSs*. Alternative transcription start sites (ATSSs) and alternative polyadenylation contribute to the mRNA diversity of *MaCCS*. ATSSs in *MaCCS* resulted in one open reading frame containing two in-frame start codons to form two protein versions, which is supported by the MaCCS subcellular localization of in both cytosol and chloroplasts. Furthermore, *MaCCS* promoter was found to contain many *cis*-elements associated with abiotic and hormonal responses. Quantitative real-time PCR analysis showed that *MaCCS* was expressed in all tested tissues (leaves, pseudostems and roots). In addition, *MaCCS* expression was significantly induced by light, heat, drought, abscisic acid and indole-3-acetic acid, but inhibited by relatively high concentrations of CuSO_4_ and under cold treatment, which suggests that *MaCCS* is involved in abiotic and hormonal responses.

## 1. Introduction

Bananas are one of the most important crops in tropical and subtropical regions. However, they are particularly susceptible to various environmental stresses, such as cold and drought, during their growth and development. Stress conditions often lead to the excessive accumulation of reactive oxygen species in cells, which results in metabolic malfunctions and cell death [1]. To reduce the damage caused by reactive oxygen species, plants employ efficient and complex antioxidative response systems, including superoxide dismutases [2]. Copper/zinc superoxide dismutase (Cu/ZnSOD), acting as a major isozyme of the superoxide dismutase family, is a dimeric copper- and zinc-containing protein that catalyzes the dismutation of superoxide radicals to protect cells from oxidative damage [3]. The zinc required for the structural stability of the Cu/ZnSOD protein can be easily obtained by passive diffusion, while the copper in Cu/ZnSOD essential for the disproportionation of superoxide can only be acquired through a copper chaperone under normal physiological conditions [4,5]. The copper chaperone for Cu/ZnSOD (CCS) specifically functions as an intracellular copper shuttle to deliver the copper to apo-Cu/ZnSOD proteins to activate them [6,7].

Although *CCS* genes have been cloned from many plants, such as *Arabidopsis* [7], tomato [8], potato [9], longan [10], poplar [11] and soybean [12], detailed analyses of *CCS* genes have only been performed in a few dicots. In addition, most previous studies focused mainly on elucidating the mechanism of action of *CCS* under conditions with limited or excess copper. Few investigations have focused on the expression and regulation of *CCS* under different types of stress. Notably, several abiotic and hormonal responsive *cis-*elements were found to be present in the promoter regions of potato *CCS* [9]. Together with the fact that the expression of *Cu/ZnSODs* was markedly induced by various stress stimuli [13] and their over-expression improved the tolerance of plants to adversity [14,15], the expression of *CCS* is likely to be affected by environmental stresses as well. Against this background, in this study, the transcriptional patterns of *CCS* under different environmental stresses were investigated in *Musa acuminata* to obtain a deeper understanding of the *CCS* gene in monocots and its role in responses to various adverse conditions.

Recently, the complete whole-genome sequences of *Musa acuminata* var. DH-Pahang (wild banana, AA genome) and *Musa balbisiana* var. Pisang Klutuk Wulang (PKW; wild banana, BB genome) were obtained [16,17], which facilitates molecular study of the *CCS* gene in bananas. Therefore, we first performed a genome-wide search for the candidate *CCS* sequences in the wild banana genomes, and then cloned and verified them in the Cavendish banana (*Musa acuminata* cv. Tianbaojiao, AAA genome). The conserved protein motifs, promoter sequence and *cis*-elements associated with stress responses of the *MaCCS* gene were analyzed to further understand its function and transcriptional regulatory mechanism. Finally, the expression patterns of *MaCCS* in response to abiotic (CuSO_4_, light, cold, heat and drought) and hormonal (abscisic acid and indole-3-acetic acid) stresses were examined, along with a comparison with the expression of *Cu/ZnSOD* genes. Analyses of the molecular characteristics and expression of *MaCCS* are helpful to understand the functions of the *CCS* gene and its collaboration with *Cu/ZnSODs* in response to different adverse conditions.

## 2. Results

### 2.1. Identification of the CCS Gene in Banana

In the wild banana genome databases, only one sequence was identified *in silico* as a *CCS* gene in “DH-Pahang” (AA genome; genome locus ID: GSMUA_Achr4G24900_001) and “PKW” (BB genome; genome locus ID: ITC1587_Bchr4_G10947), respectively. Based on the conserved regions of these two wild banana *CCS* sequences, two specific primers (CCS-ORFF and CCS-ORFR) were designed and used to amplify the open reading frame (ORF) region of the *CCS* gene in the cultivated banana “Tianbaojiao” (AAA genome). The amplified product was a 1009 bp cDNA fragment with an intact ORF of 900 bp. A BLASTp search in the NCBI database showed that it is highly similar to the CCSs from *Elaeis guineensis* (72.2% identity), *Zea mays* (60.0% identity), *Vitis vinifera* (62.6% identity) and *Arabidopsis thaliana* (54.7% identity), which suggests that it belongs to the plant *CCS* gene family. The sequence was deposited in GenBank (GenBank accession no. KM017511) and named *MaCCS*. Additionally, the ORF sequence of *MaCCS* was found to have 98.78% identity with DH-Pahang *CCS*, but only had 85.26% identity with PKW *CCS* (Figure S1).

**Figure 1:**
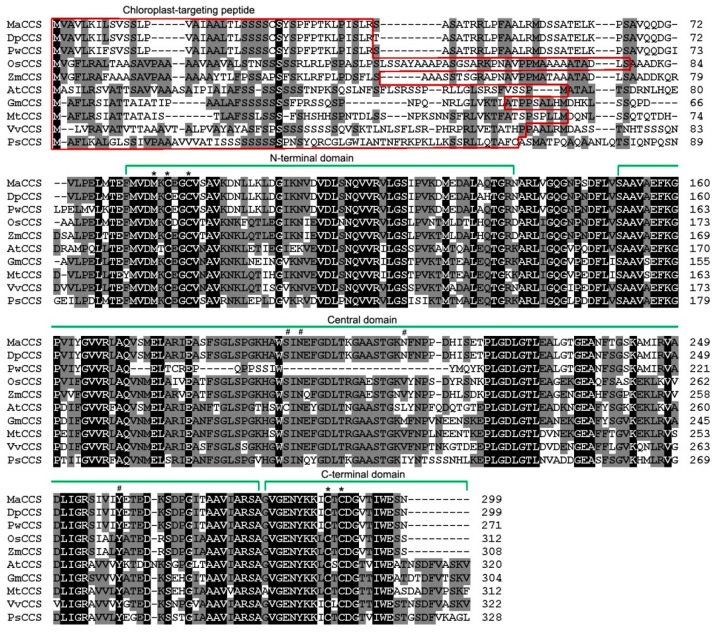
Multiple sequence alignment of the deduced MaCCS protein and other plant CCS proteins. Identical conserved residues are shown with a black background, while similar residues are indicated with a gray background. Gaps (-) have been introduced to optimize the alignment. An asterisk (*) represents the conserved metal-binding motifs (MxCxxC and CxC). A hash sign (#) indicates the replaced residues corresponding to the histidine residues of Cu/ZnSODs. The chloroplast-targeting peptides are boxed in red. The CCS proteins of Tianbaojiao, DH-Pang, PKW, *Oryza sativa*, *Zea mays*, *Arabidopsis thaliana*, *Glycine* *max*, *Medicago truncatula*, *Vitis vinifera* and *Picea sitchensis* are abbreviated as MaCCS, DpCCS, PwCCS, OsCCS, ZmCCS, AtCCS, GmCCS, MtCCS, VvCCS and PsCCS, respectively.

Protein sequence alignments showed that MaCCS possesses three typical CCS domains, just like other plant CCSs (Figure 1). The N-terminal domain of MaCCS contains a conserved metal-binding motif (MxCxxC) near the N-terminus, as previously reported in *Arabidopsis* [7], animals [18] and yeast [19], which is required for copper ion uptake when the availablity of copper is limited [7]. The central domain was shown to be homologous with its target Cu/ZnSOD proteins, which is essential for their physical interaction [20]. Three out of the four histidine residues that are copper-binding ligands of Cu/ZnSODs were previously found to be conserved in animal CCSs [8,18] but replaced by other residues in plant CCSs (Figure 1). In bananas, the copper atom is also coordinated by four histidine residues in Cu/ZnSODs (Figure S2), but, in the case of MaCCS, the four histidine residues are substituted by another four residues (Ser 194, Asn 196, Asn 211 and Tyr 259) to prevent copper binding (Figure 1 and Figure S2). The C-terminal domain of MaCCS consists of 21 residues, which is identical in number to monocot CCSs, but nine fewer than in dicot or gymnosperm CCSs. It also bears another conserved metal-binding motif (CxC), which was reported to play key roles in the interaction with the N-terminal domain to insert copper into apo-Cu/ZnSOD to activate Cu/ZnSOD [21].

### 2.2. Gene Structure and Phylogenetic Analysis of MaCCS

**Figure 2:**
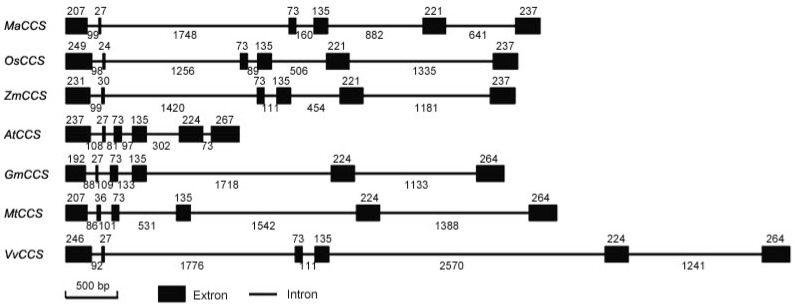
Gene structure of *MaCCS* and its homologous *CCS* genes.

To determine the exon–intron organization of *MaCCS*, a genomic DNA fragment was obtained and shown by sequencing to be 4513 bp in length (GenBank accession no. KM017509). The gene–structure map, produced by aligning the ORF gDNA with its corresponding cDNA sequence, showed that the *MaCCS* gene harbors six exons and five introns (Figure 2). By comparison with other plant *CCS* gene–structures, the *CCS* gene in bananas was shown to share the same numbers of exons and introns as those in other angiosperms. Moreover, the sizes of the third and fourth exons were conserved among angiosperms, while that of the fifth exon was conserved only between monocots/dicots, which thus should be a characteristic that can be used to discriminate monocot and dicot *CCSs*.

**Figure 3:**
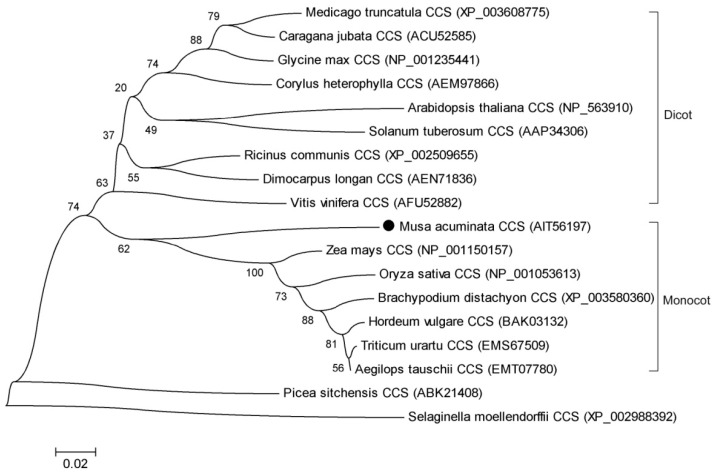
Phylogenetic tree of the *CCS* genes from bananas and other plants. The sequences were downloaded from the NCBI database and their accession numbers are given in brackets. Numbers at the nodes represent the bootstrap values based on 1000 replications.

A set of aligned sequences of MaCCS and 17 other plant CCSs retrieved from the NCBI database was used to construct a phylogenetic tree (Figure 3). Regarding the branching pattern of the phylogenetic tree, there were four distinct clusters (dicot, monocot, gymnosperm, and fern). MaCCS was grouped with other monocot CCSs in the same clade, which accords with the classified sizes of the fifth exon.

### 2.3. Subcellular Localization of MaCCS

**Figure 4:**
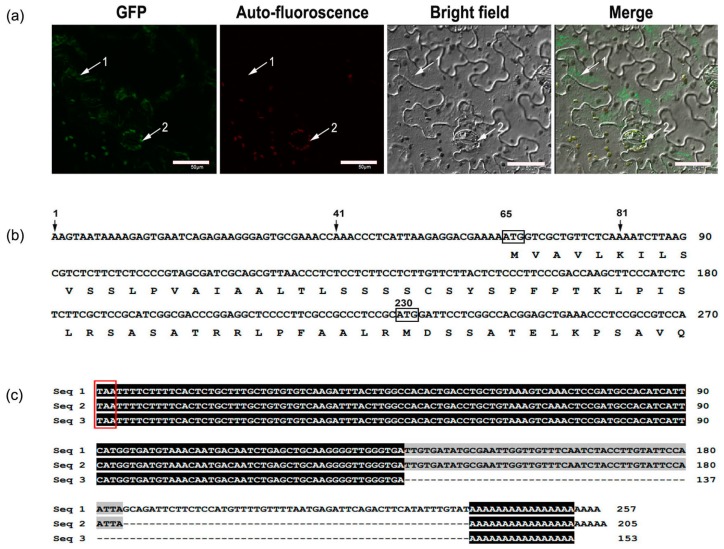
Analyses of the subcellular localization and 5′/3′ untranslated region (UTR) of the *MaCCS* gene—(**a**) Subcellular localization of MaCCS in tobacco cells. The first image presents the green fluorescence of MaCCS–green fluorescent protein under an excitation wavelength of 488 nm, the second one presents the auto-fluorescence of chloroplasts in stoma guard cells under an excitation wavelength of 561 nm, the third one presents the tobacco cells under a bright field, and the fourth one presents a merged image. Arrow 1 indicates the cytosol and arrow 2 indicates a chloroplast. Scale bar = 50 µm; (**b**) Transcription start site analysis of *MaCCS*. Arrows indicate the transcription start sites. Two in-frame ATGs are boxed; (**c**) The 3′ UTR sequence of the *MaCCS* gene. Seq. 1–3 represent three types of 3′ UTR with different lengths. The in-frame termination codon (TAA) is boxed. Nucleotides identical in all sequences are indicated with a black background, and nucleotides conserved only between two sequences are indicated with a gray background.

Signal peptide analysis using ChloroP1.1 software showed that the deduced MaCCS protein contains a chloroplast-targeting peptide like other plant CCSs (Figure 1), which agrees with the prediction results of subcellular localization obtained using the SoftBerry website. This indicates that the MaCCS protein is targeted to chloroplasts. To verify its subcellular localization, MaCCS was fused to the N-terminus of green fluorescent protein (GFP) to co-express them in tobacco leaf cells. The results showed that MaCCS was located not only in chloroplasts but also in the cytosol (Figure 4a).

### 2.4. Analysis of the Transcription Start Site and 3′ Untranslated Region of MaCCS

To understand the transcription start site of the *MaCCS* gene, a sensitive and accurate reverse transcription system (GeneRacer™ kit) was used to obtain the 5′ untranslated region (UTR) of the full-length cDNA. Ten cDNA clones obtained by 5′ RACE-PCR were randomly selected for sequencing. The results showed that the transcriptional initiation of the *MaCCS* gene could occur at three different positions (nucleotide position 1, 41 and 81) and the first nucleotide was always “A”, which resulted in one *MaCCS* ORF containing two in-frame start codons (ATG) at nucleotide positions 65 and 230 (Figure 4b). This would produce two types of peptides: the first one translated from nucleotide position 65 is 299 amino acids in length, with a chloroplastic signal peptide, and thus predicated to be located in chloroplasts, and the other one translated from nucleotide position 230 is 244 amino acids in length and is predicated to be located in the cytosol.

To obtain the 3′ UTR of *MaCCS*, ten cDNA clones produced by 3′ RACE-PCR were randomly selected for sequencing, which revealed that the *MaCCS* gene could transcribe three types of 3′ UTR with different lengths (234, 180 and 134 bp, excluding the poly-A tail), as a result of alternative polyadenylation (Figure 4c).

### 2.5. MaCCS Promoter Isolation and Cis-Element Analysis

**Figure 5:**
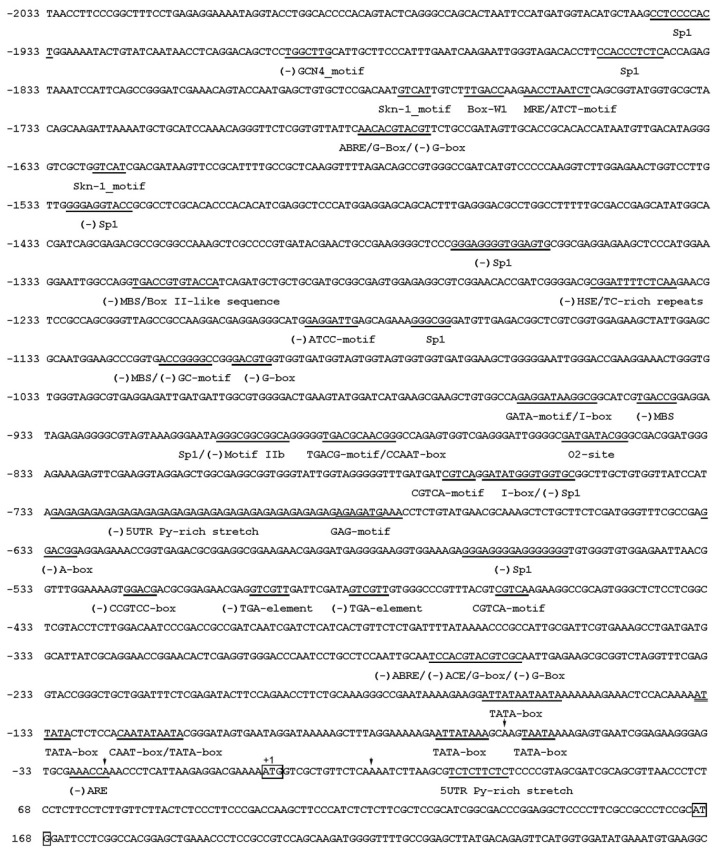
The promoter nucleotide sequence and *cis*-element analysis of the *MaCCS* gene. Motifs with high similarity to the previously identified *cis*-elements are underlined with the names given under the elements, and the reverse orientations are shown in brackets. Arrows indicate the transcription start sites. Two start codons (ATG) are shown in boxes. Coordinates give nucleotide positions relative to the first ATG site (+1).

To further elucidate the regulation mechanism of *MaCCS* gene under various stresses at the transcriptional level, a 2033-bp fragment of the 5′ flanking region from the translation start site ATG was obtained by PCR (Figure 5). Sequence analysis in PlantCARE showed that the *MaCCS* promoter harbored a typical TATA box and CAAT boxes, as well as many *cis*-elements (Figure 5).

Three types of *cis*-element were found to be abundant in the *MaCCS* promoter region. The most abundant *cis*-element class is the light responsive elements, which includes the ATCT-motif, G-box, G-Box, MRE, Sp1, ATCC-motif, GATA-motif, I box, ACE and GAG-motif [22,23,24], suggesting that the *MaCCS* gene may be regulated by light. Another abundant class of *cis*-elements in the promoter is related to hormonal responsiveness, such as ABRE and motif IIb, CGTCA-motif and TGACG-motif, and TGA-element [25,26], which are involved in responses to abscisic acid, MeJA, and auxin, respectively. The third most abundant class of *cis*-element is stress-responsive elements associating with fungal elicitor responses (Box-W1 [27]), anoxic or anaerobic induction responses (GC-motif and ARE [28]), heat stress responses (HSE [29]), and defense and stress responses (TC-rich repeats [30]). Additionally, 19 copies of a 5′ UTR Py-rich stretch, a *cis*-element conferring high transcription levels [31], were tandemly located upstream of the start codon. In summary, most of the *cis*-elements present in the *MaCCS* promoter are associated with stress and hormonal responses, suggesting that *MaCCS* is involved in the responses to various environmental stresses and hormones.

### 2.6. Expression Patterns of MaCCS under Abiotic and Hormonal Stresses

**Figure 6:**
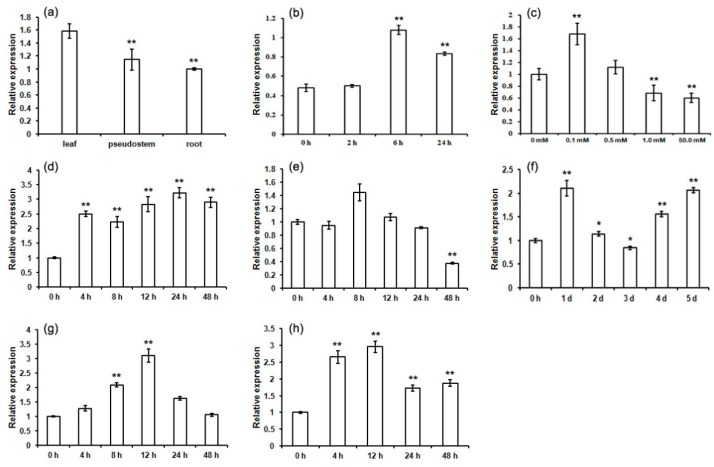
Expression levels of the *MaCCS* gene in different tissues (**a**) and under light (**b**); CuSO_4_ (**c**); heat (**d**); cold (**e**); drought (**f**); abscisic acid (**g**) and indole-3-acetic acid (**h**) treatments. Asterisks indicate significant differences from the control (** *p* < 0.01, * *p* < 0.05).

Quantitative real-time PCR (qRT-PCR) analysis showed that the *MaCCS* gene was expressed in all tested tissues, with its expression level peaking in leaves, followed by pseudostems and roots (Figure 6a). Then, the expression pattern of *MaCCS* in leaves was detected under abiotic (light, copper, heat, cold and drought) and hormonal (ABA and IAA) treatments to further understand the roles of *MaCCS* under adverse conditions. *MaCCS* expression was significantly induced by light when the leaves were exposed to light for 6 h after dark treatment (Figure 6b), revealing that *MaCCS* is regulated by light. The expression level of *MaCCS* increased in 0.1 mM CuSO_4_ treatment but decreased as the concentration of CuSO_4_ increased (Figure 6c). Under heat treatment, the expression of *MaCCS* was significantly increased at 4 h post-treatment, and >2.0-fold expression levels were maintained throughout the treatment (Figure 6d). During cold stress, the level of *MaCCS* transcripts fluctuated slightly in the first 24 h, but dramatically decreased to 0.37-fold at 48 h post-treatment (Figure 6e). Under drought treatment, the expression of *MaCCS* was dynamic, increasing to 2.1-fold on the first day, then clearly decreasing over the following two days, but finally increasing again to 2.1-fold on the fifth day (Figure 6f). In the case of ABA treatments, *MaCCS* was up-regulated in the first 12 h, with its level peaking at 3.1-fold at the 12 h point, and was then back to its original level at the 48 h point (Figure 6g). *MaCCS* was significantly induced at the transcriptional level during IAA treatment with the highest expression level of three-fold at 12 h post-treatment (Figure 6h).

## 3. Discussion

### 3.1. A Single MaCCS Gene with Diverse Transcripts

Although bananas have experienced three rounds of whole genome duplications [16], a genome-wide search for *CCS* in two wild banana genomes (AA genome and BB genome) revealed that only one *CCS* gene has been retained, indicating that the other duplicated copies were lost over the course of banana evolution, and a single copy was sufficient for functioning in bananas. Most plants, such as *Arabidopsis* and soybean [7,12], also harbor only one *CCS* gene. We experimentally cloned and verified the presence of one *MaCCS* gene in the Cavendish banana (AAA genome). *MaCCS* was shown to be more similar to the *CCS* in the AA genome than that in the BB genome, which was in agreement with the finding that the triploid *Musa acuminata* originated from crosses within diploid *Musa acuminata* [32]. The molecular characteristics of the MaCCS protein were also investigated in detail. Plant CCSs including MaCCS are diverse at the four histidine residues (copper-binding ligands of Cu/ZnSOD) in the central domain compared with animal CCSs, suggesting the divergences of *CCS* genes among flora and fauna over the course of evolution. However, MaCCS also possesses three typical domains and two conserved metal-binding motifs that are involved in the delivery of copper to apo-Cu/ZnSODs. Moreover, MaCCS exhibits a conserved evolutionary relationship with other monocot CCSs, according to the phylogenetic tree (Figure 3) and the size classification of the fifth exon (Figure 2).

Transcription of the single *MaCCS* gene could lead to several different transcripts by the use of alternative transcription start sites and alternative polyadenylation, resulting in mRNA diversity in *M. acuminate* cv. Tianbaojiao. This finding shows that there is a greater range of alternative splicing types of *CCS* genes in plants than was previously thought [12]. The distinct transcription start sites in *MaCCS* are probably involved in the production of two forms of MaCCS proteins to activate the Cu/ZnSODs in the cytosol and chloroplasts. This is supported by the subcellular localization of MaCCS in both the cytosol and chloroplasts (Figure 4a). Two versions of CCS proteins produced from a single *CCS* gene by the use of alternative transcription start sites were also detected in *Arabidopsis* [7]. This indicates that the mechanism of having a single *CCS* gene encoding two protein forms with different subcellular localizations is shared among dicots and monocots.

### 3.2. The Differential Expression of MaCCS in Different Tissues

In bananas, the expression of *MaCCS* was detected in all tested tissues, with the highest level in leaves, followed by pseudostems and roots. In a previous study on *Arabidopsis*, the *CCS* gene was found to be highly expressed in flowers but expressed at a low level in rosette and cauline leaves; at the same time, it was found to have a higher expression level in stems and flowers than in leaves at the protein level [7]. In addition, a potato *CCS* cloned by Trindade *et al.* was detected only in stem-like tissues, unlike in bananas and *Arabidopsis*, and a second homologous copy of *CCS* was found to be present in potato and to be expressed in leaves [9]. This suggests that the expression pattern of plant *CCS* in different tissues depends on the species and its copy number.

### 3.3. MaCCS Is Involved in Abiotic and Hormonal Stress Responses

A total of ten different types of light-responsive elements were found in the *MaCCS* promoter region (Figure 5), which was in accordance with the strongly up-regulated expression level of *MaCCS* under light treatment (Figure 6b), revealing that *MaCCS* participated in light response. The promoter region of *MaCCS* also carried heat-responsive (HSE) and defense and stress-responsive elements (TC-rich repeats). When subjected to heat and drought treatments, the expression of *MaCCS* was significantly increased with slight fluctuation. The up-regulation of *MaCCS* under drought was the same as previously reported for poplar *CCSs* [11]. Although *AtCCS* was found to be downregulated under heat treatment in *Arabidopsis*, the data were just obtained from one time point (without a successive course) [33]. It is known that the expression levels of abiotic stress-responsive genes exhibited dynamic change, which means that the time at which point the expression level of *CCS* is determined is critical for the results. No LTR motif (*cis*-element involved in low-temperature responsiveness) was present in the *MaCCS* promoter, and cold stress was found to inhibit the expression of *MaCCS*. This observed downregulation is similar to previous publications on bananas and plantains according to the transcriptomics data (Figure S3) [34]. In addition, *MaCCS* harbored two auxin-responsive elements in the promoter region, as well as three ABA-responsive elements, which corresponds to its increased expression under ABA and IAA treatments. Trindade *et al.* [9] also found that potato *CCS* carried three auxin-responsive elements in the promoter and its transcription was induced by auxin. These findings suggest that plant *CCS* is involved in abiotic and hormonal responses.

As the major function of CCS was reported to deliver copper to its target Cu/ZnSODs, the comparative analyses of the expression patterns between *MaCCS* and *Cu/ZnSODs* would help to further understand its roles. Regarding the correlation of expression patterns between *MaCCS* and *Cu/ZnSODs*, the levels of one or more *Cu/ZnSOD* members corresponded to that of *MaCCS* under different stress treatments using the same source of samples. Under cold treatment, *MaCCS* was obviously downregulated at 48 h, which is an expression pattern similar to those of three bananas *Cu/ZnSODs* (*MaCSD1B*, *MaCSD1D* and *MaCSD2B*) [13]. Similar correlations of expression patterns were also observed under heat stress (*MaCCS* and two *Cu/ZnSODs*: *MaCSD1B* and *MaCSD1D*) and under drought stress (*MaCCS* and two *Cu/ZnSODs*: *MaCSD1B* and *MaCSD1C*). With regard to hormone responses, *MaCCS* exhibited increased expression during 12 h under ABA and IAA treatments in bananas. Moreover, two cytosolic and one chloroplastic *Cu/ZnSODs* (*MaCSD1A, MaCSD1C* and *MaCSD2B*) also showed expression pattern highly similar to that of *MaCCS* under ABA and IAA treatment [13]. Recently, mir398 was reported to be a stress-regulated miroRNA that down-regulates the transcription of its target genes, *CCS* and *Cu/ZnSODs* (*CSD1* and *CSD2*) in *Arabidopsis* [33,35]. The binding sites for mir398 were also found in the ORF region of *MaCCS*, indicating that *MaCCS* could also be regulated by mir398 under stress conditions like *Cu/ZnSODs*. Irrespective of the abiotic stresses or hormonal treatments applied, the *MaCCS* shared transcriptional patterns consistently with its corresponding *Cu/ZnSOD*s in bananas (see our previous study [13]). This suggests that *MaCCS* and its corresponding *Cu/ZnSODs* may be regulated synchronously at the transcriptional level to ensure immediate and accurate concerted responses under adverse conditions.

## 4. Materials and Methods

### 4.1. Plant Material and Stress Treatments

Aseptic plantlets of *Musa acuminata* cv. Tianbaojiao (Cavendish banana, AAA genome) were obtained as plant materials in accordance with a previously described procedure [36]. Twenty-five-day-old plantlets were cultivated in a growth chamber at 4 °C for cold treatment and in a growth chamber at 40 °C for heat treatment. For hormonal treatment, the plantlets were cultivated in Murashige and Skoog liquid solution with 100 µM indole-3-acietic acid for IAA treatment and sprayed with 100 µM abscisic acid in 0.02% (*v*/*v*) Tween 20 for ABA treatment. Two-month-old plantlets grown in soil were cultivated without watering for drought treatment or sprayed with different concentrations (0.1, 0.5, 1.0 and 50.0 mM) of CuSO_4_ for copper stress treatment. For light treatment, the plantlets were treated under dark condition for 24 h and then exposed to light.

### 4.2. Sequence Retrieval and Gene Cloning

The whole-genome sequences of *Musa acuminata* var. DH-Pahang (AA genome) and *Musa balbisiana* var. PKW (BB genome) were downloaded from the banana genome database (http://banana-genome.cirad.fr/) [37] for a search for candidate *CCS* genes. The sequences of known *CCS* genes belonging to more than 10 plant species were also downloaded from the NCBI database, all of which were found to contain both of two characteristic motifs (motif IDs: PF00080 and PF00403) upon searching the Pfam database [38]. The whole-genome search for *CCS* genes in the two wild banana genomes was performed using Hmmer v3.0, which revealed that only one putative *CCS* gene is present in each of these genomes. Primers (Table S1) were designed to confirm its existence in *Musa acuminata* cv. Tianbaojiao (AAA genome).

DNAs, isolated using the cetyltrimethylammonium bromide method, were used as templates for gDNA and promoter cloning. Total RNAs were extracted using the Column Plant RNA_OUT_ 2.0 Kit (TIANDZ, Beijing, China) and reverse transcribed using a RevertAid First Strand cDNA Synthesis Kit (Thermo Scientific, Waltham, MA, USA) for 3′ UTR and ORF cloning, and a GeneRacer™ kit (Invitrogen, Carlsbad, California, USA) for 5′ UTR cloning and transcription start site analysis.

### 4.3. Sequence Analysis

Multiple sequence alignments of CCS proteins were performed using ClustalX v1.83 (http://www.clustal.org/). Amino acid sequences of plant *CCSs* for the alignment were downloaded from the NCBI database with the following accession numbers: OsCCS (*Oryza sativa*, NP_001053613), ZmCCS (*Zea mays*, NP_001150157), AtCCS (*Arabidopsis thaliana*, NP_563910), GmCCS (*Glycine* *max*, NP_001235441), MtCCS (*Medicago truncatula*, XP_003608775), VvCCS (*Vitis vinifera*, AFU52882), and PsCCS (*Picea sitchensis*, ABK21408). Then, their cDNA sequences and corresponding gDNA sequences were also downloaded from the NCBI database and Phytozome v10.1 (http://phytozome.jgi.doe.gov/pz/portal.html), respectively, for gene structure analysis. The gene structure map was constructed using the Gene Structure Display Sever (GSDS, http://gsds2.cbi.pku.edu.cn/). An unrooted phylogenetic tree was produced using the neighbor-joining method with MEGA 5.02 software (http://www.megasoftware.net/) [39]. *Cis*-elements in the promoter were predicted using PlantCARE (http://bioinformatics.psb.ugent.be/webtools/plantcare/html/) [40].

### 4.4. Subcellular Localization Analysis

The subcellular localization and putative chloroplastic signal sequences of *MaCCS* were analyzed using SoftBerry (http://linux1.softberry.com/) and ChloroP1.1 (http://www.cbs.dtu.dk/services/ChloroP/) [41],respectively. To confirm the subcellular localization, the *MaCCS* ORF with flanking restriction enzyme sites was amplified using specific primers (Table S1) and then ligated to the N-tersecs of the *GFP* gene in the pCAMBIA1302 vector. The recombinant vector (pCAMBIA1302-35S:*MaCCS*–*GFP*:NOS) was transformed into tobacco leaf cells using an *Agrobacterium*–mediated method [42] and then observed using a laser confocal scanning microscope (Olympus, Tokyo, Japan).

### 4.5. Expression Analysis by Quantitative Real-Time PCR

Total RNAs from different tissues and stress-treated leaves were reverse transcribed with the PrimeScript™ RT Master Mix (Perfect Real Time) kit (Takara, Shiga, Japan) for qRT-PCR analysis. Primers specific to *MaCCS* and *MaCAC* (the reference gene) [43] are listed in Table S1. qRT-PCR reactions were performed using the LightCycler480 Real-time PCR detection instrument (Roche, Rotkreuz, Switzerland) with SYBR^®^ Premix Ex Taq™ II (Tli RNaseH Plus; Takara, City Name, Japan), in accordance with the manufacturer’s protocol. Each treatment was carried out with three biological replicates and technical replicates. Relative expression levels were determined using the 2^−∆∆Ct^ method. Statistical analyses were carried out using one-way analysis of variance in SPSS v9.0 (http://www-01.ibm.com/software/analytics/spss/).

## 5. Conclusions

Banana “Tianbaojiao” (AAA genome) harbors a single *MaCCS* gene with diverse transcripts by the use of alternative transcription start sites and alternative polyadenylation. *MaCCS* shared a closer evolutionary relationship with other monocot *CCSs* than dicot *CCSs*, as well as exon-intron organizations. Sequence analysis showed that there were many light, abiotic and hormonal-responsive *cis*-elements in the promoter region of *MaCCS*, which agrees with its expression patterns under light, heat, cold, drought, ABA and IAA treatments. This suggests that *MaCCS* is involved in the abiotic and hormonal responses.

## Supplementary Materials

Supplementary materials can be found at http://www.mdpi.com/1422-0067/17/4/441/s1.

## Abbreviations

Cu/ZnSOD: Copper/Zinc superoxide dismutase
CCS: Copper chaperone for superoxide dismutase
ORF: Open reading frame
ATSSs: Alternative transcription start sites
qRT-PCR: Quantitative real-time PCR
PKW: Pisang Klutuk Wulang
ABA: Abscisic acid
IAA: Indole-3-acietic acid
NCBI: National Center for Biotechnology Information Search database
ATG: The start codon
GFP: The green fluorescent protein
5′ UTR: 5′ untranslated region
3′ UTR: 3′ untranslated region
MaCAC: Clathrin adaptor complexes medium
