# Supplementary material for: Molecular Characterization of MaCCS, a Novel Copper Chaperone Gene Involved in Abiotic and Hormonal Stress Responses in Musa acuminata cv. Tianbaojiao
Source: Int J Mol Sci. 2016 Mar 24;17(4):441. doi: 10.3390/ijms17040441 (PMC4848897; doi:10.3390/ijms17040441)
Supplement: Supplementary file 1 [file ijms-17-00441-s001.pdf]

Supplementary Materials: Molecular Characterization of *MaCCS*, a Novel Copper Chaperone Gene Involved in Abiotic and Hormonal Stress Responses in *Musa acuminata* cv. Tianbaojiao

Xin Feng, Fanglan Chen, Weihua Liu, Min Kyaw Thu, Zihao Zhang, Yukun Chen, Chunzhen Cheng, Yuling Lin, Tianchi Wang and Zhongxiong Lai

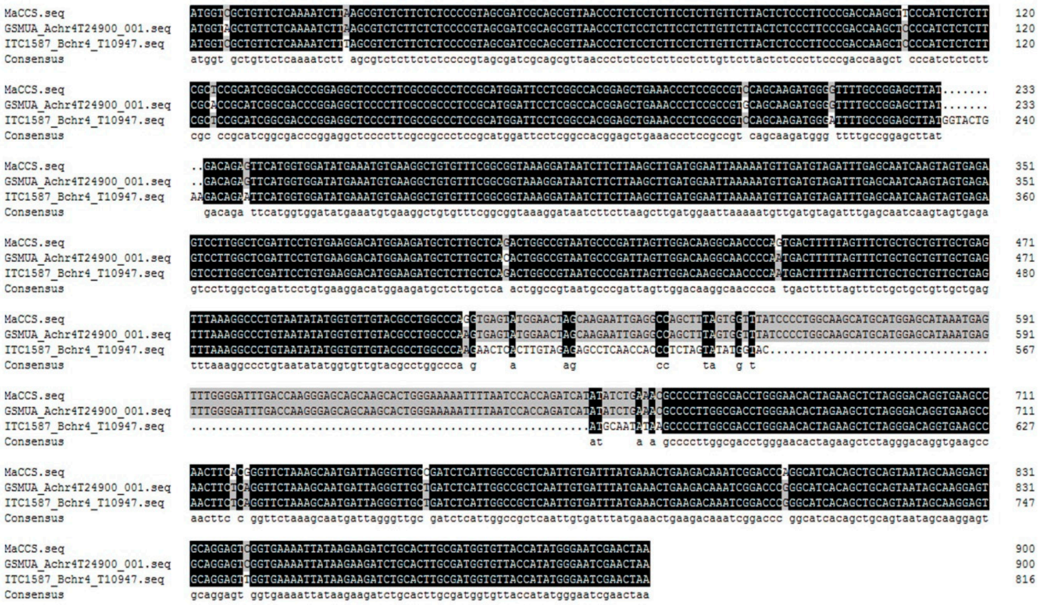

Figure S1. Sequence alignments of *CCSs* from Tianbaojiao and two wild bananas. Nucleotides identical in all sequences are indicated with a black background, and nucleotides conserved only between two sequences are indicated with a gray background.

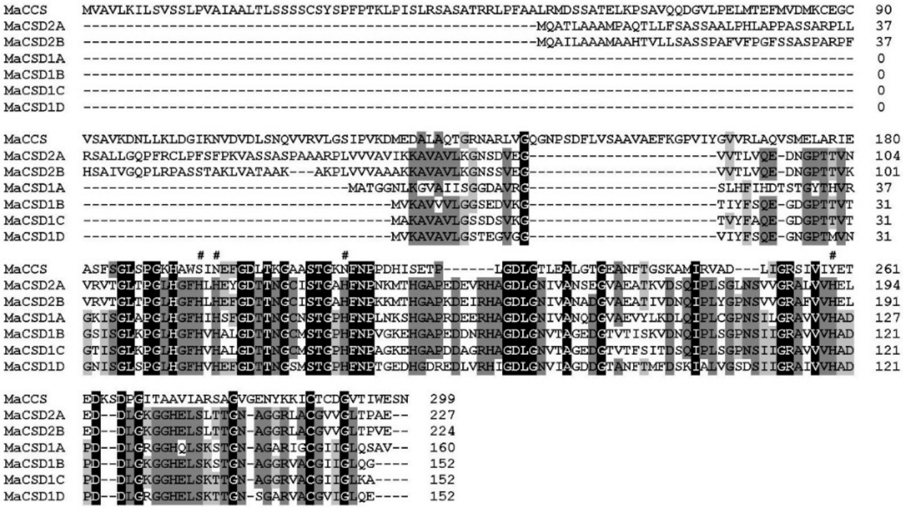

Figure S2. Multiple sequence alignment of the deduced MaCCS protein and Cu/ZnSOD (MaCSD) proteins. Identical conserved residues are shown with a black background, while similar residues are indicated with a gray background. Gaps (-) have been introduced to optimize the alignment. A hash sign (#) indicates the copper-binding ligands (histidine residues) of Cu/ZnSODs. Amino acid sequences of the MaCSDs are downloaded from the NCBI database with the following accession numbers: MaCSD2A (AFV46365), MaCSD2B (AIF32069), MaCSD1A (AFC17494), MaCSD1B (AGC13157), MaCSD1C (AGC13158) and MaCSD1D (AGC85069).

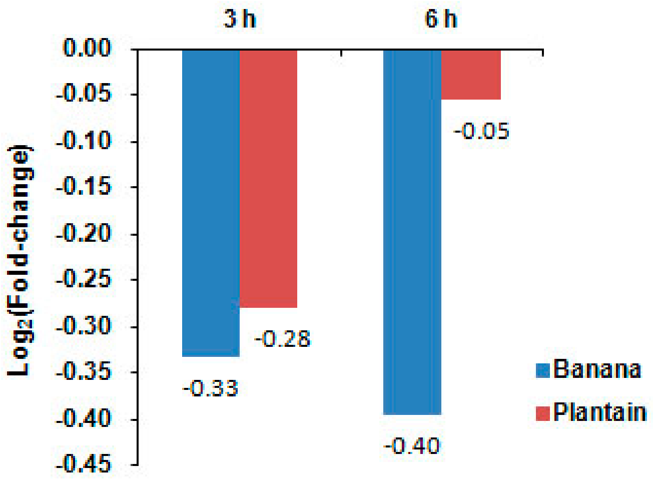

Figure S3. Expression levels of the *CCS* gene (genome locus ID: GSMUA_Achr4G24900_001) in response to cold treatment (10 °C) according to the transcriptomics data. The expressions of *CCS* are presented using fold change compared to control (0 h). Log_2 (fold-change value) is shown at the top of bar.

Table S1. Oligonucleotide primers used in this study.

Primer Name	Primer Sequence (5′–3′)	Tm/°C	Extension Time/s	Purpose
CCS-ORFF<br>CCS-ORFR	GCAAGTAATAAAAGAGTGAATCGGAG<br>CTTTACAGCAGGTCAGTGTGG	54	55	ORF/gDNA amplification
CCS-5R1<br>5P	CACTAAAGCTGGCCTCAATTCTTGC<br>CGACTGGAGCACGAGGACACTGA	58	35	1st round of 5′RACE
CCS-5R2<br>5NP	GCCAAGGACTCTCACTACTTGATTGC<br>GGACACTGACATGGACTGAAGGAGTA	60	30	2nd round of 5′RACE
CCS-3F1<br>AUAP	GAGCAATCAAGTAGTGAGAGTCCTTG<br>GGCCACGCGTCGACTAGTAC	58	50	1st round of 3′RACE
CCS-3F2<br>AUAP	CTAGCAAGAATTGAGGCCAGCTTTAG<br>GGCCACGCGTCGACTAGTAC	60	40	2nd round of 3′RACE
CCS-ProF<br>CCS-ProR	GAACATGAGCATCCTGATAAAGTG<br>AAGAGGAGAGGGTTAACGCTG	55	120	Promoter amplification
CCS-SubF<br>CCS-SubR	CATG**CCATGG**ATGGTCGCTGTTCTCAAAATC *<br>GG**ACTAGT**GTTCGATTCCCATATGGTAAC *	51	54	Subcellular localization
CAC-QF<br>CAC-QR	AACTCCTATGTTGCTCGCTTATG<br>GGCTACTACTTCGGTTCTTTCAC	57	30	qRT-PCR
CCS-QF<br>CCS-QR	GAAGATGCTCTTGCTCAGACTG<br>GGCTAGTTCCATACTCACCTGG	57	30	qRT-PCR

* Restriction enzyme sites are in bold.
